# Early EEG and NIRS measurements in preterm babies: a systematic review

**DOI:** 10.1007/s00431-024-05712-2

**Published:** 2024-08-07

**Authors:** R. Llamas-Ramos, J. J. Alvarado-Omenat, I. Llamas-Ramos

**Affiliations:** 1 Department of Nursing and Physiotherapy, Universidad de Salamanca, Avd. Donantes de Sangre s/n, 37007 Salamanca, Spain; 2 IBSAL) and Primary Care Research Unit of Salamanca (APISAL), Biomedical Research Institute of Salamanca, Salamanca, Spain; 3 FisioSport Salamanca, S.L, Salamanca, Spain; 4 University Hospital of Salamanca, Salamanca, Spain; 5 Health Service of Castile and Leon (SACyL), Salamanca, Spain

**Keywords:** EEG, NIRS, Assessment, Preterm

## Abstract

Preterm birth represents a public health problem, with prematurity being the leading cause of infant mortality. An objective brain maturation and oxygenation measurement are necessary. The objective has been To test the feasibility of EEG and NIRS combination in the assessment of physiological brain maturation and oxygenation in preterm and non-preterm babies. A systematic review in Pubmed, Web of Science, MEDLINE, Cochrane, Dialnet, CINAHL, Scopus, Lilacs and PEDro databases until December 2022 was developed. 598 registers were found, finally 5 of them reached the inclusion criteria. Two independent reviewers analyzed data and a third reviewer were available for discrepancies. All articles combined EEG and NIRS to assess brain oxygenation and maturation in healthy new-born babies. There is an agreement on the electrode’s placement for EEG at P3 and P4; besides, these regions have been shown to be a development predictive area, as well as the frontoparietal region for the NIRS region and comparison between regions. There is little evidence about the physiological brain electrical activity and oxygenation without stimuli.

*Conclusion*: EEG and NIRS have been useful to assess brain electrical activity and oxygenation in preterm and non-preterm. The combined measurement of these instruments could be essential in neurological disorders diagnosis or their sequels. Unfortunately, the heterogeneity of the results found prevents a consensus on which variables are the most appropriate for the assessment of this population.

**Table:** 

What is Known	
*• Brain assessment could help clinicians to prevent sequels.*	
*• There is an agreement for EEG electrodes placement at P3 and P4 region.*	

What is New	
*• EEG and NIRS assessment are effective measurements for preterm babies.*	
*• P3 and P4 regions have shown to be a predictive area of development, as well as the frontoparietal region for NIRS assessment.*

**Supplementary Information:**

The online version contains supplementary material available at 10.1007/s00431-024-05712-2.

## Introduction

Preterm birth continues to be a public health problem, with prematurity being the leading cause of infant mortality [1–3]. The rate of prematurity is increasing considerably in recent years [4], although it is true that most of these deliveries are usually spontaneous, there are more and more iatrogenically induced preterm deliveries to avoid possible complications such as preeclamsia or gestational diabetes [5]. Fortunately, recent advances in neonatal care have increased survival rates in this population [6, 7].

The WHO divides preterm birth according to gestational age into moderate or late preterm (32 to 37 weeks), very preterm (28 to 32 weeks) and extreme preterm (gestational age less than 28 weeks) [8]. The immaturity of the brain in premature infants makes them more vulnerable to the development of lesions, which can lead to neurological deficits [9].

The risk of developing a neonatal adverse effect is estimated to be 7 times higher in premature infants than in term infants [10]. In addition, in the case of very premature infants, it has been shown that they may present structural anomalies in the brain anatomy [11, 12]. Among the possible adverse effects that premature infants may suffer, there are multiple short-term complications [13, 14], and in the long term, these premature infants may experience cognitive and motor consequences and even altered academic performance, with lower intelligence quotient than full-term infants [2, 15, 16]. A meta-analysis published by Aarnoudse-Moens et al. [17] reflects the possible deficits related to preterm birth.

To all these complications must be added an increased risk of hospital admissions and neurological deficits [18] and the enormous social and economic costs involved [19], with the average number of days of hospitalization estimated at between 4 and 135 days [20, 21].

There are several methods for objective assessment and measurement of these effects. The most common are electroencephalography (EEG) and near infrared spectography (NIRS). EEG measures brain electrical activity, which will represent brain function, and brain oxygenation or brain metabolic demand will be measured by NIRS. This technology began in the 1970s with the goal of monitoring oxygenation in living tissues [22] and has proven to be a feasible, noninvasive, and beneficial technology [23, 24]. Several studies support the use of both instruments [24].

The measurement of both parameters is important since they are related, even changes in oxygenation precede changes in EEG [24]. In addition, it has also been shown that cerebral metabolic demand and oxygen supply can be disrupted in pathologies or be an indicator of them [25, 26].

More and more authors are indicating the need for early rehabilitation treatments to try to reduce or even eliminate these possible complications experienced by premature infants [27], which makes a correct evaluation of the baseline situation of this population necessary.

The aim of the present study was to test the feasibility of the combination of EEG and NIRS in the measurement of brain maturation and oxygenation that occurs in preterm and non-preterm infants.

## Materials and methods

This systematic review has been previously registered at PROSPERO with the ID: CRD42022302448 and the PRISMA recommendation statement has been followed [28].

### Systematic literature research

Bibliographic research has been carried out in several databases: Pubmed, Web of Scicene, MEDLINE, Cochrane, Dialnet, Lilacs, ProQuest, Scopus, PEDro, and EBSCO from January 2022 to December 2022. The search terms used were “preterm”, “neonat”, “cortical activation”, “brain activation”, “measure”, “evaluation”, “EEG”, and “NIRS”. No search limits were established. Language has been limited to English, French, and Spanish, and PICO strategy has been followed:

- Population: newborn babies (term and preterm) without pathologies.
- Interventions: EEG and NIRS measurements.
- Comparisons: parameters fluctuations in the same group of babies during the assessment.
- Outcomes: cerebral maturation and/or oxygenation have been the main variable measured.

### Selection criteria

This systematic review included studies following the next inclusion criteria: [1] healthy term or preterm newborns without significant additional comorbidities; [2] articles that measure brain activation and brain oxygenation through EEG and NIRS technology without stimuli. The exclusion criteria were as follows: (1) articles in adults, (2) invasive measurement techniques, (3) associated pathologies (epilepsy, sepsis, ventricular dilatations, hypothermia, encephalopathy, spinal cord injury, asphyxia, schizophrenia, stenosis, paralysis, stroke, cerebral palsy, autism, syncope, syncope), (4) childbirth complications, (5) no full text found, (6) theoretical articles based in this technology, and (7) other types of publication as reviews, cases, editorials or letters to editor have not been considered.

### Screening, selection, and data extraction

Two reviewers screened the potential articles independently (R. L-R and I. L-R). Duplicate articles were removed at the beginning of the screening. In the next step, the title and abstract to select the eligible articles were considered by the reviewers. After that the selected articles were full text read. Besides, a third reviewer (J.J. A-O) was available if there is any disagreement until a consensus was reached.

Both reviewers (R. L-R and I. L-R) extracted the main data from the selected articles: authors, year, sample, variables, procedure, results, and conclusion. The third reviewer was also available to obtain a consensus if there were discrepancies.

### Assessment of methodological quality and risk of *bias*

To assess the quality of each study analyzed independently, the JBI (Joanna Briggs Institute) scale for cross-sectional analytical studies was used. This scale consists of 8 items and a maximum score of 8 points. A score of 8 points means high quality; a score of 5 to 7 means moderate quality; a score of 3 to 4 means low quality; and a score of 2 or less is interpreted as very low quality (Annex 1).

## Results

### Study selection

**Fig. 1:**
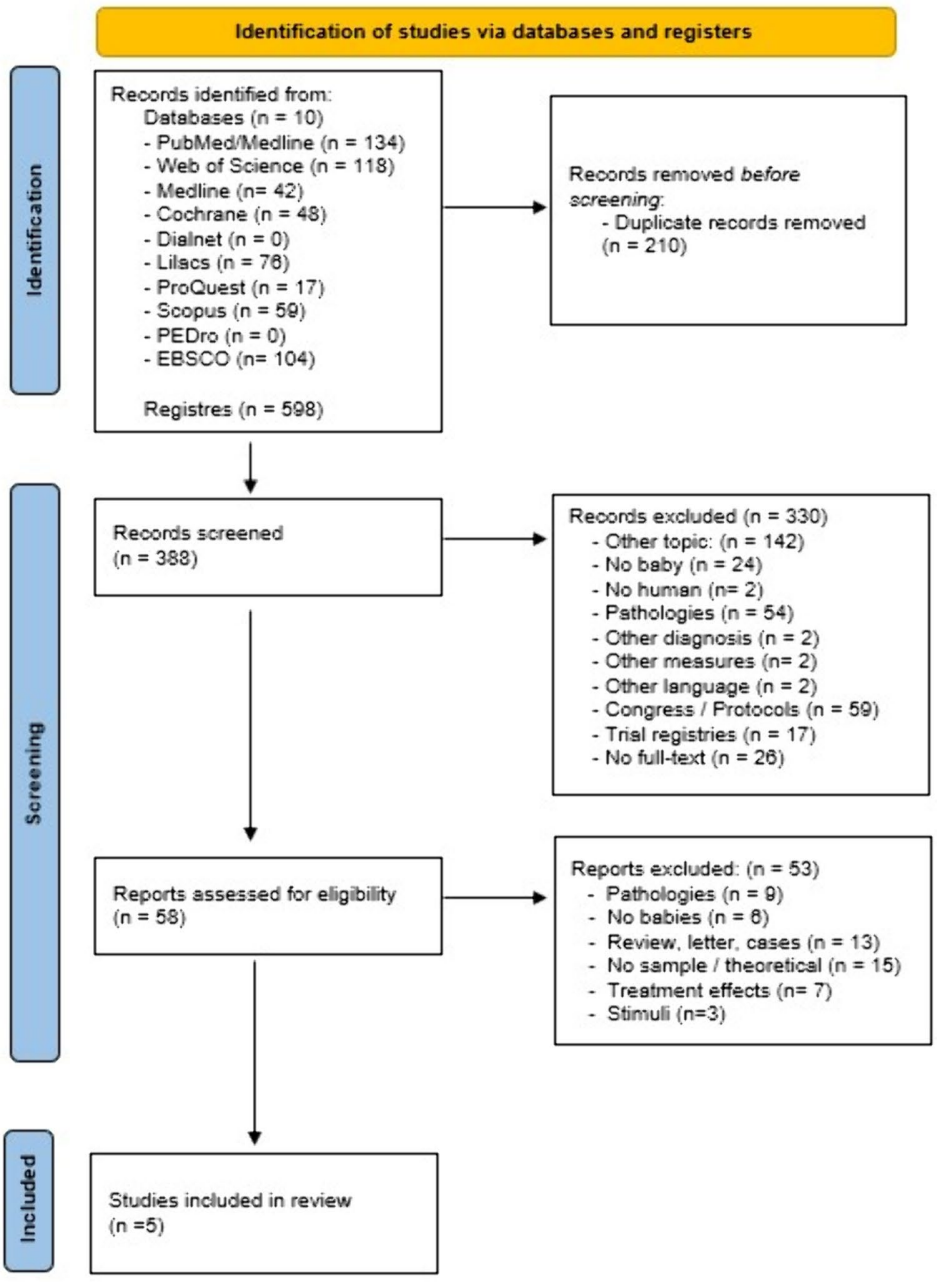
PRISMA flowchart of article selection

Five hundred ninety-eight articles were identified as a potential paper for inclusion on this manuscript. 210 duplicated files were removed. After title and abstract screening, the sample size was reduced in 388 files. Fifty-eight articles obtained after the title and abstract screening were full-text reading by two independent reviewers. At the end, five manuscripts following the established inclusion and exclusion criteria composed the final sample. Figure 1 shows the PRISMA flowchart of article selection.

A summary was made of the most relevant characteristics of each of the articles included (Table 1).

**Table 1:** Summary of the main characteristics of the selected articles

***Author/year***	***Sample***	***Variables***	***Procedure***	***Results/conclusions***
*Roche-Labarbe *et al*. (2007) *[29]	6 preterm	HbO_2_, HHb, and HbT	EEG and NIRS measurement. In incubator with a cloth over it to eliminate all external stimuli. EEG: 8 electrodes plus one ground electrode NIRS: 2 emitters and a detector over the left temporal area	Recording time 68 ± 14 min EEG bursts in the T3 channel. A pattern of deoxygenation, followed by oxygenation associated with the onset of the EEG burst is observed. It was clearer for HbO_2_ than for Hhb There is evidence of spontaneous physiological neuronal activity in premature infants combined with a hemodynamic stereotyped pattern
*Tataranno *et al*. (2015) *[30]	44 preterm	rScO_2_, cFTOE, brain activity measurements like SAT, SAT rate, ISI, and minimum aEEG	Monitoring from 3 h after birth, selecting a recording between 4 and 6 h after birth 2 EEG channels (P3 and P4) and 2 NIRS sensors (frontoparietal)	Evaluation time: 50 ± 8 min. Mean blood pressure: 32 ± 6 mmHg. Minimum and maximum Pco2: 30 and 60 mmHg. Brain activity changed significantly with gestational age. rScO_2_ did not change with gestational age and was negatively associated with SAT and minimum EEG and positively associated with ISI
*El-Dib *et al*. (2016) *[31]	46 very low birth weight preterm	Absolute and relative powers of EEG (delta, theta, alpha, beta, total frequency bands) and FTOE	EEG: 6 electrodes placed in C3, C4, P3 and P4 region to generate 4 evaluation channels (P3-P4, C3-C4, C3-P3 and C4-P4) NIRS: adhesive sensor and pulse oximeter	Evaluation time: 21.3 ± 5.5 min FTOE (short scale): positive correlation with the relative power of the delta band (*r* = 0.45) and negative correlation with the relative power of the alpha and beta bands (*r* = − 0.38 and *r* = − 0.29). There was no significant correlation with the relative power of the theta band (*r* = − 0.2) FTOE (short scale): positive correlation with the relative power of the delta band (*r* = 0.44) and negative correlation with the relative power of the alpha band (*r* = − 0.42 and *r* = − 0.29). There was no significant correlation with the relative power of the theta band (*r* = − 0.21) and beta band (*r* = − 0.25) The more mature the EEG activity, the less variable the FTOE
*Tamussino *et al*. (2016) *[32]	9 study group 50 control group	aEEG, crSO_2_, and cFTOE	Monitoring at the first moment of birth (cesarean section) Four regions C3 and C4 (parietal) and FP1 and FP2 (frontal) were selected In addition pulsi oximeter in right wrist	Monitoring: 15 min after delivery Maximum and minimum volumes were lower in the study group than in the control group. FTOE: was higher in the study group than in the control group (after 10 min they were equal). Saturation was lower in the study group than in the control group (at minute 7 the difference was significant)
*Ter Host *et al*. (2011) *[33]	46 preterm babies	rcSO_2_, FTOE, and aEEG	Simultaneous measurement of EEG and NIRS in the first 24 h after birth, at 2, 3, 4, 5, 8, and 15 days EEG: 2 neonatal electrodes at P3 and P4 NIRS: frontoparietal region Pulsi oximeter	There were no changes in rScO2, FTOE and postnatal age from day 1 to 5. There were significant changes between the 5th and 15th day (decreased from 79 to 70%) FTOE: increase from 0.16 to 0.20 from day 5 to 8 and from 0.20 to 0.26 between day 8 and 15 There is a relationship between brain activity and oxygen consumption. If activity matures, oxygen consumption increases and oxygen consumption is influenced by postnatal age and hemoglobin levels The combination of FTOE and electrocerebral activity may be a biomarker of brain function in high-risk children

*aEEG* amplitude-integrated EEG, *EEG* electroencephalography, *NIRS* near infrared spectography, *rScO2* regional cerebral oxygen saturation, *crSO*_*2*_ cerebral regional oxygen saturation, *pCO2* pressure of CO2, *(c)FTOE* (cerebral) fractional tissue oxygen extraction, *SAT* number of spontaneous activity transients, *ISI* SAT intervals, *HbO*_*2*_* and HHB* concentrations of oxy- and deoxy-hemoglobin, *HbT* total hemoglobin

In all the articles included in the present review, oxygenation, and brain electrical activity have been evaluated by NIRS and EEG, respectively, in healthy newborn infants without pathology. In all of them, the diagnostic usefulness of the combination of both instruments has been demonstrated [29–33].

Regarding the sample, the studies presented a very variable sample size from 4 babies [29] to 46 babies [31, 33]; however, there was a consensus in the placement of electrodes at P3-P4 level and surface electrodes at frontoparietal level to obtain cerebral oxygenation, while some authors used 2 electrodes [30, 33], others 6 electrodes [31] and other authors 8 electrodes [29]. In addition, all measurements were performed in the first hours of life of the infants, from birth [32] to 15 days with simultaneous measurements from 24 h after birth [33].

First, Labarbe et al. [29] evaluated the physiological discontinuous activity of newborn premature infants during sleep to test the existence of a relationship between changes in oxy- and deoxyhemoglobin concentration and the onset of spontaneous bursts of electrical activity. These authors confirmed that the decrease in deoxyhemoglobin concentration occurs a few seconds before the onset of electrical activity. In addition to healthy children, they evaluated separately children with pathology (respiratory distress) confirming that there are differences in NIRS parameters between them.

Tataranno et al. [30] also used the combination of NIRS and EEG to assess changes in brain metabolism early (first 6 h after birth). In this way, they postulated that oxygen supply and consumption is directly related to brain functional activity expressed by transient spontaneous activity in the first hours after birth showing an increase in oxygen extraction.

El-Dib et al. [31] evaluated the fractional cerebral tissue oxygen extraction (FTOE) together with the pulsi-oximeter to test whether in very low birth weight infants the more mature the EEG activity, the less variable the FTOE. They concluded that increased maturation of EEG activity is associated with decreased variability of cerebral oxygen extraction.

Tamussino et al. [32] also evaluated the relationship between brain activity and regional cerebral oxygen saturation in neonates at immediate delivery, as well as the relationship between FTOE and brain activity. Their results were that low brain activity at the initial transition with low oxygen saturation occurred, but also increased oxygen extraction.

Ter Host et al. [33] state that an alteration in oxygen supply can cause brain damage in premature infants. The objective was to test the relationship between brain tissue oxygen saturation, FTOE, and brain activity. They showed that an increase in FTOE is accompanied by a more mature brain activity.

The methodological quality of the articles was that four papers were of moderate quality [29–31, 33] and one manuscript showed of low quality [32]. No papers show high quality (Table 2).

**Table 2:** JBI scale for the quality of cross-sectional analytical studies

*Article- item*	*Roche-Labarbe *et al*. *[29]	*Tataranno *et al*. *[30]	*El-Dib *et al*. *[31]	*Tamussino *et al*. *[32]	*Ter Host *et al*. *[33]
*Were the sample inclusion criteria clearly defined?*	Y	Y	Y	Y	Y
*Were the study subjects and environment described in detail?*	Y	Y	Y	C	Y
*Was the exposure measured in a valid and reliable way?*	Y	Y	Y	Y	Y
*Were standard and objective criteria used to measure the condition?*	NA	NA	NA	NA	NA
*Were confounding factors identified?*	N	N	Y	N	N
*Were strategies in place to address confounding factors?*	N	N	Y	N	N
*Were the results measured in a valid and reliable way?*	Y	Y	Y	Y	Y
*Was an appropriate statistical analysis used?*	Y	Y	Y	Y	Y
*TOTAL*	5	5	7	4	5

*Y* yes, *N* no, *C* confusing, *NA* no aplicable

The great heterogeneity of the variables evaluated in the different articles prevents us from establishing comparisons.

Roche-Labarbe et al. [29] concluded that there is evidence of spontaneous physiological neuronal activity in premature infants combined with a hemodynamic stereotyped pattern like El-Dib et al. [31] who found that the more mature the EEG activity, the less variable the FTOE; however, for other authors [30] brain activity can change by gestational age while the rScO2 do not have to change which means that there is no relation between these variables. Finally, Ter Host et al. [33] in other study showed that the combination of FTOE and electrocerebral activity may be a biomarker of brain function in high-risk children. This last conclusion is of special relevance given the fragility of this population, being necessary an early diagnosis to avoid sequelae.

## Discussion

Combination of EEG and fNIRS assessment has demonstrated to be feasible and could be a predictor of a potential sequel that preterm could develop. Besides, brain electrical activity has correlation with hemodynamic patterns.

Most studies index the neurovascular response through cognitive stimuli or pathological conditions such as seizures [34]; therefore, in the present review, we selected articles without any stimulus that could influence the results. A knowledge of the conditions of oxygenation and physiological brain maturation in newborns can be very useful to define the typical pattern facilitating the development of a model that helps to better understand the complete function of the infant brain. It would be a useful tool for clinicians to prevent, diagnose, and prescribe early treatments thus avoiding neurological and developmental problems early to avoid sequelae, since according to some authors in children with pathology the hemodynamic pattern may be weak and less efficient, and in premature infants the pattern is also different due to the immaturity of the brain [29].

The effectiveness of the combination of EEG and NIRS has been widely evidenced in recent years, showing a relationship between brain maturation and oxygen consumption. EEG and NIRS have proven to be effective and reliable in measuring brain electrocerebral activity and oxygenation in infants. Specifically, NIRS may be a useful biomarker for brain vulnerability in high-risk infants [30]; moreover, increased variability in FTOE in brain injury in preterm infants needs further investigation [31]. Low levels of cerebral perfusion and oxygen concentration may imply altered brain electrocerebral activity [33]. Besides, many authors have investigated these measurements in response to different stimuli. Specifically, Telkemeyer et al. [35] investigated the brain response of 3- and 6-month-old infants in relation to speech perception divided into fast acoustic modulations and slow modulations. Their results showed that fast modulations stimulated bilateral neural activations, whereas slow modulations generated right-lateralized responses, underlining the importance of these responses in language acquisition. Coinciding with the previous authors, Cabrera et al. [36] assessed speech perception by measuring the ability of neonates to process acoustic speech signals and demonstrated that EEG responses indicate that neonates can encode consonants in all conditions, even without fast temporal modulations, like adults. However, fast and slow temporal modulations activate different neural areas, as shown by NIRS. This leads to the conclusion that the immature human brain is already capable of decomposing the acoustic components of speech, laying the foundation for language learning [36]. Along the same lines, other authors investigated the response of newborns to acoustic stimuli. They demonstrated a cerebral blood change in volume followed by an increase in oxyhemoglobin and hemoglobin concentrations due to increased oxygen utilization in the homolateral temporal cortex of newborns and suggest a possible frontotemporal cerebral pathway for storing unusual sounds [37]. Similarly, there are reviews that have tested the effectiveness of these assessment procedures in isolation, this is the case of Pavlidis et al. [38] and Kong et al. [39] both teams investigated EEG brain measurement, exposing the continuity, sleep state, synchrony, and transient waveforms presented by this population; however, the results are too heterogeneous to demonstrate which EEG characteristics are the best in the cognitive aspect. Regarding NIRS, Kooi et al. [40] concluded that there is a lack of consensus on which NIRS signals should be used to optimize the measurement of brain autoregulation. These results agree with those obtained in the present review where the heterogeneity of the tested studies prevents obtaining conclusive results.

This monitoring has been shown to be useful in identifying children at risk of developing hemorrhages [35, 41, 42] since monitoring, the hemodynamic responses of neuronal activation, are not fully developed in the neonatal brain but there is a weekly maturation of brain activity [43–46]. There is agreement on the placement of electrodes in the EEG at P3 and P4, as these regions have been shown to be a predictive area of development [47], as well as the frontoparietal region for the NIRS region and comparison between regions [48, 49].

In the present review, to avoid variables that could interfere with the results, studies with infants presenting pathology such as respiratory distress syndrome were excluded, where it was shown that an acute increase in end-tidal CO_2_ is associated with increased cerebral oxygenation and decreased brain electrical activity, while an acute decrease is associated with decreased cerebral oxygenation and a slight increase in brain electrical activity [50]. Finally, Biallas et al. [51] evaluated the responses of 14 term neonates to an optical stimulus during sleep to test the sensitivity and repeatability of NIRS to detect hemodynamic response, as well as the sensitivity and repeatability of EEG. Concluding very good results for NIRS and verifying if inadequate stimulation could be the reason for the absence of hemodynamic responses.

It should be noted that Labarbe et al. [29] established 2 groups (healthy and with pathology) that were analyzed independently. In the present review, the data from the healthy group have been considered. These authors demonstrated that there are differences in the parameters evaluated, confirming the hypothesis of the existence of differences in the presence of brain injury.

Among the limitations of the present review is the wide variability of the studies found, there being no homogeneity in the procedures and methodology that would allow comparison; in the same way that this limitation has prevented the performance of a meta-analysis given the existing differences between the outcome variables and units of measurement selected by the authors, age of the children and even pathologies they presented, which could interfere in the results.

The strict inclusion criteria have severely limited the final sample. It is believed that the condition of no stimuli application can generate a more objective evaluation of the changes that occur at the level of oxygenation and brain maturation without the external influence of a stimulus in this population. Unfortunately, the search for articles whose sample was composed of healthy newborns or preterm infants has been small.

In future lines of research, the clinical effect of interventions or treatments to stimulate brain activation and oxygenation, boosting brain development and therefore avoiding neurological alterations and their subsequent sequelae in premature infants will be tested.

## Conclusion

The combination of simultaneous measurement of NIRS and EEG has proven to be reliable and suitable for assessing cerebral oxygenation and brain maturation in premature infants. Measurement parameters of brain electrical activity and oxygenation are related and may be indicative of brain injury. Unfortunately, the heterogeneity of the results found prevents a consensus on which EEG and NIRS variables are the most appropriate for the assessment of this population and more studies are needed.

## Electronic supplementary material

Below is the link to the electronic supplementary material.

Supplementary file1 (DOCX 31 KB)

## Data Availability

No datasets were generated or analysed during the current study.
